# Subconjunctival Ab Externo Gel Stent Implantation for Refractory Glaucoma After High-Risk Penetrating Keratoplasty

**DOI:** 10.7759/cureus.8873

**Published:** 2020-06-27

**Authors:** Lanxing Fu, Michelle L Baker, Fiona Carley, Leon Au

**Affiliations:** 1 Department of Cornea, Manchester Royal Eye Hospital, Manchester, GBR; 2 Department of Glaucoma, Manchester Royal Eye Hospital, Manchester, GBR

**Keywords:** cornea, glaucoma, gel stent implant, xen, xen45, penetrating keratoplasty, transplantation, ab externo

## Abstract

This case study reports the successful deployment of the XEN45 gel stent (AbbVie Inc, Chicago, IL) through an ab externo approach in a 73-year-old woman with refractory glaucoma following high-risk penetrating keratoplasty (PK) 10 years prior. The PK was for corneal perforation secondary to peripheral ulcerative keratitis, which required systemic immunosuppression comprising intravenous cyclophosphamide, azathioprine, and corticosteroids to stabilise the disease and prevent corneal graft rejection. The patient’s intraocular pressure was reduced from 40 mmHg preoperatively to 12 mmHg six months after surgery, off medication. The patient’s visual acuity and visual fields remained stable. The XEN45 gel stent utilising the ab externo approach can be considered as a potential tool to lower intraocular pressure in patients with glaucoma after corneal keratoplasty.

## Introduction

Glaucoma is the leading cause of visual loss after keratoplasty [1-3]. High intraocular pressure (IOP) can cause a reduction of corneal endothelial cells, graft failure, and damage to the optic nerve [1,2]. The risk of glaucoma developing is higher in the presence of associated ocular surface inflammation, corneal perforation, combined procedures, angle distortion, and compression [1]. 

The 6-mm XEN45 gel stent (AbbVie Inc, Chicago, IL) traditionally utilises an ab interno approach, forming a channel for aqueous to flow from the anterior chamber into the subconjunctival or subtenon space [4]. The stent is hydrophilic and composed of gelatin cross-linked with glutaraldehyde, allowing it to be non-degrading and without a foreign body reaction. 

This case report describes the alternative ab externo subconjunctival implantation technique of the XEN45 stent in a patient with refractory glaucoma following a high-risk penetrating keratoplasty performed 10 years ago. 

## Case presentation

**Figure 1:**
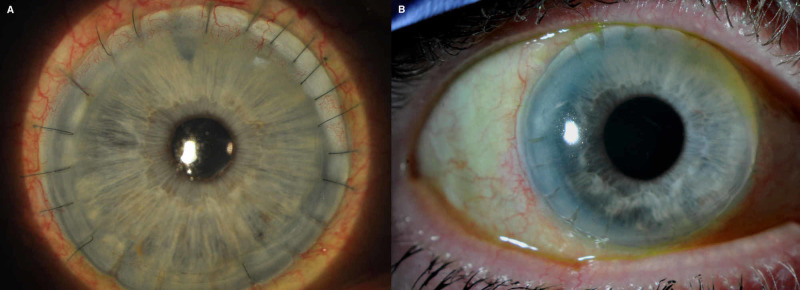
Large diameter penetrating keratoplasty at 12 months postoperative (A) and at 10 years (B) for complications of peripheral ulcerative keratitis.

A 73-year-old woman presented to her routine glaucoma follow-up review with high IOP. Her past ocular history included secondary glaucoma following a large diameter (limbus-to-limbus) penetrating keratoplasty with concurrent lens extraction and intraocular lens implant for corneal perforation due to peripheral ulcerative keratitis (Figure 1). The disease was poorly controlled, necessitating a six-month course of intravenous cyclophosphamide as per the EuroLupus protocol, followed by a tapering regime of oral azathioprine and prednisolone [5]. Prior to presenting with high IOP, the patient had not been on systemic immunosuppression for three years. Secondary glaucoma developed following graft surgery and had been managed with topical latanoprost and dorzolamide hydrochloride/timolol maleate eye drops. Her past medical history included osteoporosis and spinal fractures, which developed as a consequence of corticosteroid treatment and irritable bowel syndrome. 

On examination at presentation, her vision was decreased at 0.5 logMAR (logarithm of the minimum angle of resolution) best corrected in the right eye, compared with 0.1 logMAR three months prior. Goldmann applanation tonometry measured IOP of 40 and 14 mmHg in right (OD) and left (OS) eyes, respectively. Slit-lamp examination revealed no anterior chamber activity and a well-centred “in-the-bag” intraocular lens implant. Gonioscopy showed an open angle but view of the angle structures was suboptimal due to distortion from the graft-host interface of the large-diameter corneal graft. Optic nerve examination revealed increased vertical cup to disc ratios of 0.9 and was corroborated with visual fields that had evidence of progression.

As the IOP was poorly controlled with maximal tolerable topical medications and there was documented visual field progression, surgery to lower IOP was recommended. An XEN45 gel stent was implanted in the right eye. A subconjunctival ab externo approach was chosen in light of the poor angle view and to avoid the potential endothelial damage to the corneal transplant from surgical manipulation within the anterior chamber.

Video 1 demonstrates the ab externo implantation of the gel stent implant. The superior conjunctiva was marked at 2.5 mm from the limbus at 12 o’clock. Subconjunctival injection of 0.1 mL of lignocaine 2% to create a subconjunctival space for XEN implantation followed by 0.1 mL of 0.2 mg/mL mitomycin C (MMC) adjacent to the target area was applied. This was kept away from the limbus with an anteriorly placed squint hook. The XEN45 injector was introduced into the subconjunctival space from the superior fornix in a manner similar to the standard bleb needling technique. Caution was taken to ensure the injector needle stayed above any tenon tissue while being advanced towards the limbus. Once the tip of the injector needle reached the 2.5-mm mark near the limbus, it was then directed into the sclera, tunnelling it parallel to the scleral plane, entering the anterior chamber. When the tip of the needle was just visible in the anterior chamber, the blue slider of the injector was then advanced halfway to partially deploy the XEN45. In order to avoid an excessive amount of XEN45 being implanted into the anterior chamber, the injector was withdrawn while the slider was advanced through the second half of its travel. Once the deployment was complete, the injector was retracted out of the subconjunctival space. The external subconjunctival portion of the XEN45 was checked for good mobility and length (3 mm). The ideal dimensions for XEN45 stent insertion were adhered to, with 1 mm in the anterior chamber, 2 mm tunnelled through the sclera, and 3 mm exposed in the subconjunctival space. A bleb was formed immediately on the operating table, and subconjunctival injection of antibiotics and dexamethasone was given.

**Video 1:** Subconjunctival ab externo gel stent implantation.

**Figure 2:**
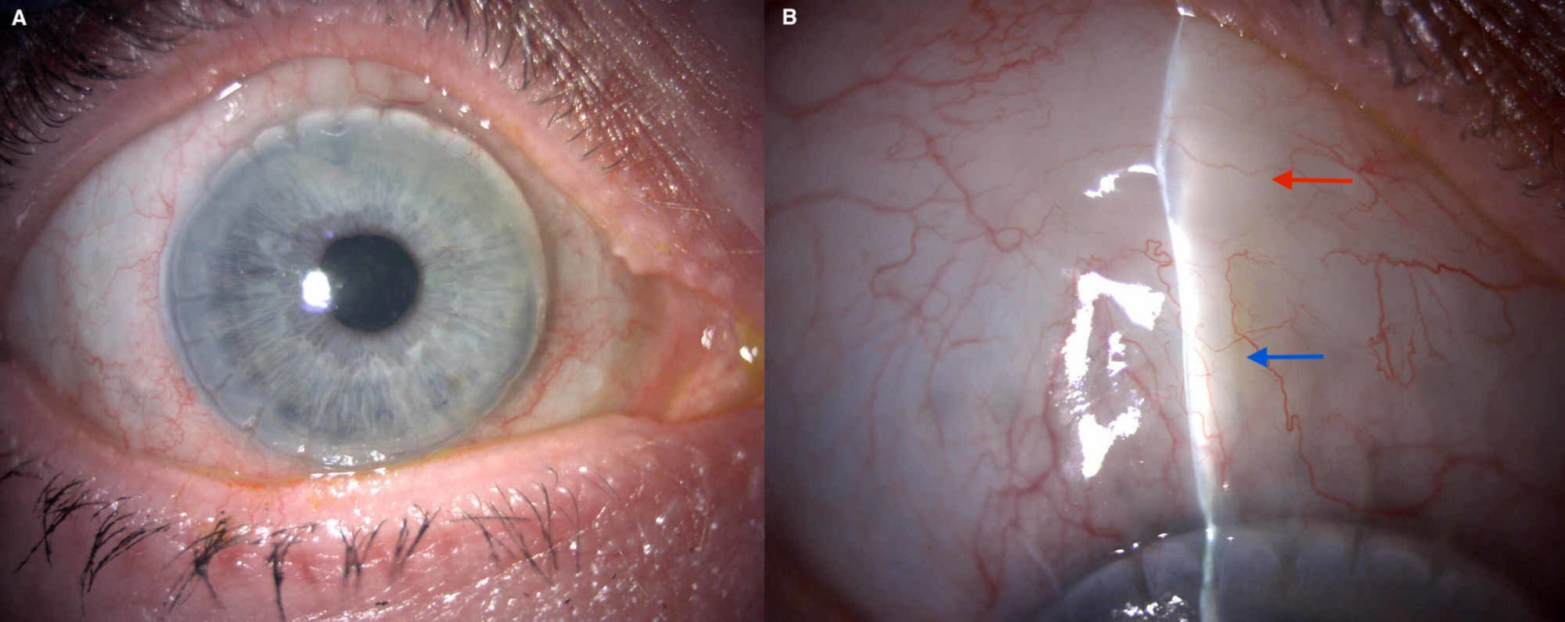
Appearance of the right eye six months postoperative XEN45 implantation. Panel A shows a clear corneal graft. The curvature of the light reflex superiorly is consistent with the formation of a posteriorly positioned bleb (panel B, red arrow pointing to bleb); the XEN45 stent is visible in the subconjunctival space at 12 o'clock (panel B, blue arrow pointing to gel stent implant).

On the first postoperative day, IOP measured 8 mmHg with Goldmann applanation tonometry with a diffuse bleb, deep anterior chamber, and normal fundus examination. In particular, the corneal graft was clear and there was no anterior chamber activity. The patient was commenced on two hourly topical dexamethasone, tapering over a three-month period to once a day for corneal graft maintenance. Topical chloramphenicol 0.3% was given for two weeks. Postoperative bleb management comprised of one 5 mg 5-fluorouracil subconjunctival injection at week 2 due to increased bleb vascularisation. The patient developed an episode of herpes simplex keratitis which resolved with prompt treatment of topical and systemic antivirals. IOP was 12 mmHg on no glaucoma medication following XEN45 implantation with a clear corneal graft and visual acuity of 0.12 logMAR, posteriorly placed bleb, and subconjunctival gel stent implant at three months postoperatively (Figure 2). At last follow-up (nine months) after gel stent implantation, the visual acuity was maintained, IOP was 13 mmHg, and visual fields remain stable. The patient remained on topical dexamethasone 0.1% once daily for corneal graft maintenance.

## Discussion

The total number of corneal transplants performed annually has increased globally in recent years [2,3]. Glaucoma after corneal transplant is multifactorial, and contributory causes include corticosteroid use, synechial angle closure, iatrogenic injury to the angle, vitreous prolapse, inflammation, and retained viscoelastic [1]. In our patient, the size of the graft, which involved the angle, and the prolonged use of steroids in the postoperative period were likely contributing factors leading to secondary glaucoma. The first-line treatment for raised IOP in this group of patients remains topical glaucoma therapy, which the patient managed with for a decade with no adverse effect. Selective laser trabeculoplasty was discounted due to her distorted angle anatomy from a large limbus-to-limbus cornea transplant; cyclodestructive procedures were undesirable due to the induced postoperative inflammation, disruption to the ocular surface, and the risk of exciting cornea graft rejection. Trabeculectomy will have a guarded outcome due to previous ocular surgery and inflammation.

Glaucoma drainage devices (GDD) are considered a good choice in the management of refractory post-keratoplasty glaucoma [6,7]. Tube placement can be in the anterior chamber, sulcus, or vitreous cavity depending on whether the patient is phakic, pseudophakic, or had previous pars plana vitrectomy, with good IOP control at up to five years postoperatively [6]. However, the incidence of graft failure is high. Almousa et al. found the percentage of corneal grafts remaining clear at one, two, three, and five years to be 87%, 77%, 65%, and 47%, respectively, in their cohort of 59 eyes with Ahmed valve implantation [8]. There is also a lack of consensus regarding the ideal implant type, tube location, and timing of GDD insertion (concurrent or consecutive). Ayyala et al. compared outcomes of trabeculectomy with MMC, GDD, and cyclophotocoagulation in 38 patients and found no significant difference with respect to controlling IOP and graft failure [9]. However, the presence of a trabeculectomy bleb may preclude patients from utilising future contact lens correction.

Minimally invasive glaucoma surgery (MIGS) has emerged in recent years as a new class of glaucoma procedure with proposed advantages of a high safety profile, faster surgical times, and rapid postoperative recovery [10]. Several MIGS procedures are now available including the iStent (Glaukos Corporation, San Clemente, CA), InnFocus MicroShunt (Santen Pharmaceutical Co., Ltd, Osaka, Japan), and the XEN45 implant. Their smaller size potentially makes them less prone to endothelial damage in comparison to GDDs although conclusive endothelial data have yet to be published on new devices like the XEN45 gel stent. We believe that the use of the XEN45 in our case would lead to less surgical trauma, especially to the corneal transplant. The implantation technique was modified to the ab externo subconjunctival approach, as described above, to avoid surgical manipulation within the anterior chamber. This technique avoids the need for a corneal incision and cohesive viscoelastic (as in the traditional ab interno approach). This technique also avoids conjunctival peritomy, diathermy, and suture closure used in the ab externo open conjunctival approach described by Grover et al [11]. This new approach further reduces surgical time and offers immediate IOP reduction which was required in this patient with a high IOP despite maximal medical therapy. The patient had good preoperative spectacle-corrected visual acuity, which allowed for a bleb forming drainage procedure without the fear of contact lens interference. 

There have not been previously reported cases of a XEN45 stent in patients with refractory glaucoma in high-risk post-penetrating keratoplasty, with an ab externo approach. The XEN45 is akin to traditional glaucoma filtration surgery, except without the need for conjunctival manipulation, a scleral flap, ostium, or suturing. The unique advantages of the XEN45 implant were more appropriate in this patient as it allowed for immediate IOP lowering and reduced conjunctival manipulation and inflammation, thereby possibly reducing the risk of future graft rejection. Furthermore, the posterior placement of the bleb also allowed for potential future contact lens use if necessary for visual rehabilitation. Rahmania et al. reported good outcomes with XEN45 implantation in a series of five patients after corneal graft surgery using the conventional ab interno technique [12]. This technique required a good view of the anterior chamber with gonioscopy, which was not possible in our case. Concurrent gel stent implantation at the time of corneal graft surgery can theoretically reduce the procedural numbers and the need for long-term topical medication. However, the postoperative rehabilitation can be prolonged, with subsequent effect on graft survival. Therefore, the timing of the surgery will depend on the type of corneal graft performed and the mechanism of raised IOP, which will dictate the choice of MIGS implant.

## Conclusions

This case demonstrates that an ab externo XEN45 stent is an effective treatment option that can be added to the glaucoma surgeon’s armamentarium for the management of glaucoma in a high-risk postpenetrating keratoplasty eye. Further studies are necessary to assess the long-term efficacy, safety, and surgical timing of the XEN45 in the management of refractory glaucoma in patients with corneal grafts.
